# Immunomodulating and Immunosuppressive Therapy for Virus-Negative Immune-Mediated Myocarditis

**DOI:** 10.3390/biomedicines12071565

**Published:** 2024-07-15

**Authors:** Andrea Frustaci, Claudio Letizia, Maria Alfarano, Giulia Marchionni, Romina Verardo, Cristina Chimenti

**Affiliations:** 1 Cellular and Molecular Cardiology Laboratory, IRCCS Lazzaro Spallanzani, 00149 Rome, Italy; romina.verardo@inmi.it; 2 IRCCS San Raffaele, 00163 Rome, Italy; 3 Department of Clinical, Internal, Anaesthesiology and Cardiovascular Sciences, Sapienza University of Rome, 00161 Rome, Italy; claudio.letizia@uniroma1.it (C.L.); maria.alfarano@ymail.com (M.A.); cristina.chimenti@uniroma1.it (C.C.); 4 Policlinico San Matteo Pavia IRCCS Foundation, University of Pavia, 27100 Pavia, Italy; giulia.marchionni01@universitadipavia.it

**Keywords:** myocarditis, autoimmunity, immunosuppressive therapy, endomyocardial biopsy

## Abstract

Myocarditis is an inflammatory disease of the myocardium caused by infectious and noninfectious agents. Clinical manifestations range from mildly symptomatic forms to acute heart failure, cardiogenic shock, life-threatening arrhythmias and sudden death. Myocarditis is still a challenging diagnosis because of its wide variability in clinical presentation and unpredictable course. Moreover, a standardized, specific treatment in not yet available. Immunosuppressive treatment for virus-negative lymphocytic myocarditis is still controversial. Conversely, immunosuppression is well established in sarcoidosis, eosinophilic, giant-cell, drug hypersensitivity, and trauma-related myocarditis as well as lymphocytic myocarditis associated with connective tissue diseases or with the rejection of a transplanted heart. Recently, immunosuppressive therapy has been also recognized as an effective treatment in virus-negative inflammatory cardiomyopathy. The aim of this review is to underline the role of immunomodulating and immunosuppressive therapies in patients with immune-mediated myocarditis and illustrate the different treatment strategies depending on the etiology. An endomyocardial biopsy remains the gold standard for the diagnosis of myocarditis as well as for a tailored treatment.

## 1. Introduction

Myocarditis is an inflammatory disease of the myocardium that is diagnosed by established histological, immunological, and immunohistochemical criteria [1]. The pathogenesis of myocarditis is complex, and it is mainly related to three causes: infections (viruses, bacteria, and fungi), systemic immune-mediated diseases, and toxins (drugs, vaccines, toxic agents), with viruses being the prevalent etiological agent in Western countries [2]. The incidence is 4 to 14 people per 100,000 each year globally [3]. The risk is major among young people between 20 and 40 years old and in the male sex [4]. Clinical manifestations range from mildly symptomatic forms to acute heart failure, cardiogenic shock, life-threatening arrhythmias, and sudden death [5]. Myocarditis spontaneously resolves in approximately 50% of cases; however, in 25% of cases, it determines persistent left ventricular dysfunction, and in the 12–25% of cases, it may evolve into end-stage heart failure [6]. The prevalence of myocarditis in young people who die suddenly ranges from 2 to 42% [7]. In a prospective registry of Northeastern Italy, the incidence was 12% [8].

Myocarditis is still a challenging diagnosis because of wide variability in clinical presentation and unpredictable course. In the last two decades, the diagnostic workup has been implemented with the introduction of cardiac magnetic resonance imaging (MRI) [9,10]. As a consequence, a diagnosis of “clinically suspected myocarditis” can be made on the basis of non-invasive diagnostic tools: clinical presentation, electrocardiography, structural and functional assessment of echocardiography, and tissue characterization of cardiac MRI. According to the 2007 American Heart Association (AHA)–American College of Cardiology (ACC)–ESC scientific statement [11] and more recent expert consensus documents [12,13], EMB is recommended in the case of clinically suspected myocarditis with severe clinical manifestation including cardiogenic shock, acute heart failure requiring inotropes, mechanical circulatory support, ventricular arrhythmias, or high-degree atrioventricular blockage. Moreover, it is also indicated in autoimmune disorders with progressive heart failure that are unresponsive to treatment with/without sustained ventricular arrhythmias and/or conduction abnormalities and suspected immune checkpoint inhibitor (ICI)-mediated myocarditis. In acute myocarditis, as previously documented, cardiac MRI sensitivity is high for infarct-like presentation, low for cardiomyopathic presentation, and very low for arrhythmic clinical presentation [14]. Therefore, although cardiac MRI is a useful diagnostic tool, EMB still remains the gold standard for diagnosis, and it is the basis for appropriate and effective treatment.

Patients with myocarditis should be treated with optimal care in the case of heart failure and arrhythmias according to the current guidelines [15,16,17], regardless of etiology. Conversely, disease-specific treatment, which has a prevalent role in immune-related forms, requires EMB with histologic and immunohistochemical characterization and a molecular biology search for viral genomes.

**Figure 1:**
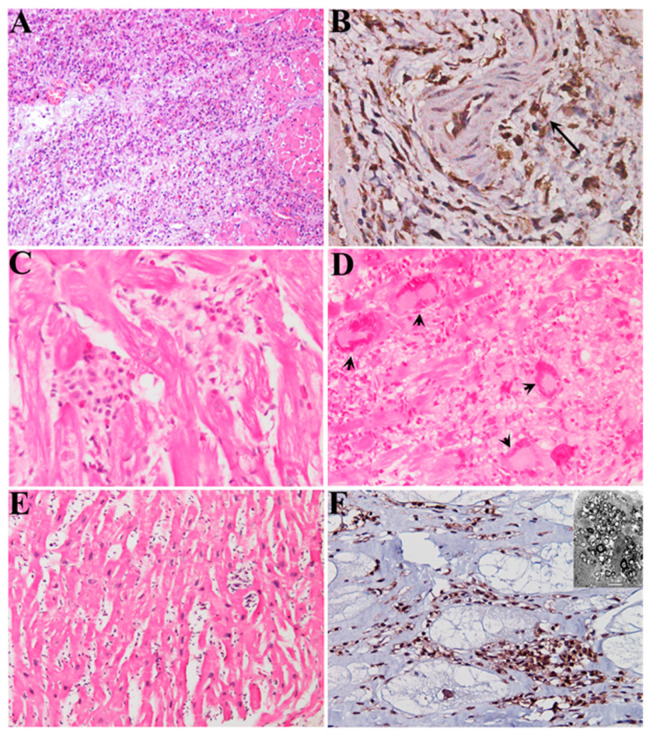
**Major types of myocarditis that are amenable to immune suppressive therapy.** (**A**) Virus-negative lymphocytic myocarditis. H&E 100×. (**B**) Lymphocytic myocarditis associated with necrotizing coronary vasculitis. Immunostaining with CD45Ro, 400×. (**C**) Eosinophilic myocarditis. H&E 325×. (**D**) Giant cell myocarditis. H&E 200×. (**E**) Hypertrophic cardiomyopathy with over-imposition of lymphocytic myocarditis. H&E 225×. (**F**) Fabry disease cardiomyopathy with over-imposition of immune-mediated (vs. GB3) myocarditis. Immunostaining with CD45Ro, 400×. Scale bar, 5 microns.

The aim of this review is to underline the role of immunomodulating and immunosuppressive therapies in patients with immune-mediated myocarditis (Figure 1) and to illustrate the different treatment strategies depending on the etiology.

## 2. Immunosuppressive Therapy in Virus-Negative Lymphocytic Myocarditis: Evidence from the Literature

In 1995, Mason et al. investigated the efficacy of a 24-week treatment with prednisone and either cyclosporine or azathioprine in addition to standard therapy versus conventional therapy alone in active myocarditis in a myocarditis treatment trial (MMT) [18], showing no benefits for myocardial function. This study had major limitations, as myocarditis was diagnosed only on the basis of the Dallas criteria at histology without immunohistochemical characterization of the inflammatory infiltrates, and it mainly did not distinguish between viral and non-viral forms. In 2001, Wojnicz et al. [19] showed that in patients with myocarditis and up-regulation of HLA antigen in the myocardial tissue, a 3-month treatment with prednisone and azathioprine determined improvements in the left ventricular (LV) ejection fraction (EF), reductions in the LV diastolic dimension and volume, and reductions in the New York Heart Association (NYHA) functional class in 71.8% of treated patients versus 20.9% of patients in the placebo group. Even if in this study the presence of viral genome in the myocardium was not assessed, the increase in HLA expression could be considered an indicator of susceptibility to immunosuppression. 

For the first time, our group, based on both retrospective and prospective studies, identified the characteristics of patient responders to immunosuppressive therapy and the cellular and molecular mechanisms of cardiac recovery after immunosuppression. In a retrospective study, 41 patients with active lymphocytic myocarditis and chronic heart failure (HF) lasting over 6 months were evaluated. They were all treated with immunosuppressive therapy, including prednisone 1 mg/kg/day for 4 weeks followed by 0.33 mg/kg/day for 5 months and azathioprine 2 mg/kg/day for 6 months. The patients were classified as responders if they had a decrease in at least one NYHA class and an improvement in EF ≥ 10% compared to the baseline measures [20]. Among the 41 patients, 21 had significant improvements in LVEF and healed myocarditis based on the control biopsy. Twenty patients were classified as non-responders and showed a histological evolution toward dilated cardiomyopathy; twelve remained stable; three underwent cardiac transplantation; and five died. Retrospective PCR performed on frozen endomyocardial samples and evaluation of circulating cardiac autoantibodies in the patients’ sera showed that the non-responders had a high prevalence of viral genomes in the myocardium (85%) and no detectable autoantibodies in the serum, whereas 90% of the responders were positive for autoantibodies, with only three (15%) presenting viral genomes based on the PCR analysis. These three responder patients were all positive for hepatitis C virus, so the beneficial effects of immunosuppression in hepatitis C virus myocarditis may be related to an immune-mediated mechanism of damage. 

To confirm these results in a prospective manner, a randomized, double-blind, placebo-controlled, single-center trial was performed, enrolling patients with active lymphocytic myocarditis and chronic heart failure with no evidence of viral genomes in the myocardium based on a PCR analysis. Among the 85 patients, 43 patients (Group 1) were treated with prednisone 1 mg/kg/day for 4 weeks followed by 0.33 mg/kg/day for 5 months and azathioprine 2 mg/kg/day for 6 months, and 42 patients (Group B) were treated with a placebo for 6 months in addition to conventional HF therapy [21]. Group 1 showed a significant improvement of LVEF and a decrease in left-ventricular dimensions and volumes compared with the baseline (88% of patients); even patients with severe left ventricular dilation (LV end-diastolic diameter up to 90 mm) and dysfunction (LVEF < 20%) had a prompt response to immunosuppression. All of the Group 2 patients showed LVEF reductions compared with the baseline. None of the patients on immunosuppression had major drug-related side effects requiring therapy withdrawal. Control histology at 1 and 6 months showed healed myocarditis with the disappearance of inflammatory infiltrates and interstitial and focal replacement fibrosis in the responder patients of group 1. Control biopsies of the Group 2 patients revealed the persistence of myocarditis as well as the expansion of interstitial and replacement fibrosis. In 2022, a 20-year follow up of the TIMIC trial was published, confirming the lasting benefits of immunosuppression in this population, including in terms of LV function, the need for ICD implantation, or heart transplant and death [22]. 

In this study, the long-term clinical outcomes of 85 patients (including the entire population of the TIMIC trial, since the placebo group was also switched to a 6-month immunosuppressive therapy at the end of the study period) were compared with those of a 1:2 propensity score-matched control group of myocarditis patients (Group B). At long-term follow-up, the risk of cardiovascular death and heart transplantation was significantly higher in Group B patients, as well as the need for implantable cardioverter defibrillator implantation. Group A showed a persistent improvement in the left ventricular ejection fraction. The incidence of recurrent myocarditis was similar between the groups, and patients with evidence of a recurrent cardiac inflammatory process promptly responded to a TIMIC protocol application. Similarly, a study from Escher et al. reported the 10-year follow-up of patients with virus-negative myocarditis treated prednisone and azathioprine for 6 months, confirming the long-lasting benefits of IS, with resolution of the myocardial inflammation based on histology [23]. 

A recent meta-analysis confirmed the efficacy of immunosuppression in terms of lower mortality and improved cardiac function in biopsy-proven myocarditis with chronic HF [24]. Currently, a multicenter randomized study (IMPROVE-MC EudraCT: 2020-003877-23) on immunosuppression in virus-negative myocarditis is ongoing [25]. One hundred patients with biopsy-proven myocarditis and reduced LVEF (>45%) have been randomized to a 12-month treatment with prednisone and azathioprine (according to the TIMIC protocol) or a placebo in addition to optimized medical therapy. The study will also assess the persistence of therapy effects after a subsequent 12-month observation period. In a recent study, Caforio et al. documented that prolonged tailored immunosuppressive therapy (median duration of 19 months, QR 10–26) is effective and safe in biopsy-proven immune-mediated myocarditis [26].

In the setting of fulminant myocarditis, the recent AHA Scientific Statement [27] suggests the possibility of administering high-dose intravenous glucocorticoids (1 g of methylprednisolone) in patients with cardiogenic shock, ventricular arrhythmias, or high-degree atrioventricular block before a biopsy-confirmed diagnosis. In this context, The Myocarditis Therapy with Steroids (MYTHS) trial (ClinicalTrials.gov identifier: NCT05150704) is ongoing, assessing the efficacy of pulsed intravenous high-dose corticosteroid therapy (1 g intravenous methylprednisolone daily for 3 days) versus a placebo in addition to conventional therapy in patients suspected of acute myocarditis complicated by cardiogenic shock/acute heart failure.

Alternative therapies that are commonly used in pediatric patients with lymphocytic myocarditis include intravenous immunoglobulin (IVIG) [28], but the data in adults are limited [29]. The Anakinra vs. Placebo for the Treatment of Acute Myocarditis (ARAMIS) trial (ClinicalTrials.gov identifier: NCT03018834) is a double-blind randomized clinical trial evaluating the efficacy and safety of Anakinra, an interleukin-1 receptor antagonist, in addition to standard care in patients with acute myocarditis throughout the 28 days after hospital discharge [30]. The results, presented during the ESC Congress 2023, show that Anakinra is safe but does not reduce complications. CardioMyoPathy With MYocarditis THerapy With Colchicine (CMP-MYTHiC) is an ongoing, randomized, single-blinded, multi-center, phase III controlled trial with two parallel groups: patients with chronic inflammatory cardiomyopathy administered colchicine (1 mg daily or 0.5 mg daily il weight < 70 kg) versus patients receiving a placebo for 6 months (ClinicalTrials.gov Identifier: NCT06158698).

## 3. Immunosuppressive Therapy in Virus-Negative Lymphocytic Myocarditis Associated with Systemic Immune-Mediated Diseases

Lymphocytic myocarditis can be associated with systemic immune-mediated diseases (SIDs), including autoimmune and inflammatory diseases [31]. Clinical presentation is unspecific and includes unexplained dyspnea, palpitations, chest pain with or without increased troponin, syncope, arrhythmias, acute or chronic heart failure, and cardiogenic shock. Fever is also a comment manifestation. Cardiac MRI and positron emission tomography (PET) are useful non-invasive diagnostic tools to detect myocardial inflammation, but they can only differentiate between infectious and non-infectious forms, allowing for safe immunosuppressive treatment. Myocardial involvement is common in patients with systemic lupus erythematosus (SLE): myocarditis treatment in these patients includes pulsed intravenous high-dose methylprednisolone followed by oral corticosteroids in association with azathioprine, mycophenolate mofetil, cyclophosphamide, or intravenous immunoglobulin [32]. Myocardial inflammation in systemic sclerosis is associated with a poor prognosis, and it responds to immunosuppression [33]. Patients with myocarditis and catastrophic antiphospholipid antibody syndrome (CAPS) must be treated with anticoagulation, high-dose corticosteroids, and either IVIG or plasma exchange [34]. Rituximab can be used in association with plasma exchange [35]. Myocarditis associated with eosinophilic granulomatosis with polyangiitis (EGPA, formerly Churg–Strauss syndrome) has adverse outcomes [36] since it may lead to restrictive cardiomyopathy or dilated cardiomyopathy. Unexplained heart failure in patients with polyarteritis nodosa can be related to immune-mediated myocarditis and intramural vessel vasculitis, immunosuppressive treatment with corticosteroids and cyclophosphamide allows for cardiac recovery [37]. The beneficial effects of immunosuppression in patients with lymphocytic virus-negative myocarditis and associated necrotizing coronary vasculitis have also been demonstrated [38], as well as in patients with immune-mediated myocarditis and pemphigus, gouty, psoriasis, and Fabry cardiomyopathy, where myocardial inflammation may contribute to disease progression and resistance to enzyme replacement therapy [39]. Alternative therapies in immune-mediated myocarditis include the removal of circulating autoantibodies through immunoadsorption and IVIG [40,41,42].

## 4. Markers of Susceptibility to Immunosuppression

Biological markers that are able to predict the response to immunosuppression in myocarditis patients are extremely useful in clinical practice. The current literature mainly focuses on serological and immunohistochemical markers: the first are represented by circulating anti-heart autoantibodies (AHA) that are disease-specific and present in 60% of myocarditis patients [43]. They recognize multiple cardiac antigens, particularly cardiac α-myosin heavy-chain and β-myosin heavy-chain isoforms [44], and they may have a direct pathogenic or prognostic role in immune-mediated myocarditis and dilated cardiomyopathy [45]. The presence of AHA in sera may be used as a marker to identify patients who can benefit from immunosuppression [46]. The immunohistochemical markers are represented by myocardial over-expression of human leucocyte antigen (HLA)-DR [47] and toll-like receptor 4 (TLR4). Toll-like receptors (TLRs) regulate the innate immune system in the induction and perpetuation of inflammation in autoimmune diseases [48]. TLR4 is implicated in the development of several experimental and human autoimmune disorders [49]. In the heart, TLR4 binds the endogenous ligand following cardiac injury, and it is an important mediator of inflammatory reactions [50]. It has been demonstrated that semiquantitative evaluation of immunostaining (grades from 0 to 4) for TLR4 showed increased cardiomyocyte expression in myocarditis patients responding to immunosuppression [51]. Gradings of 2 or above (2+) at baseline showed a sensitivity of 100%, a specificity of 90.9%, a positive predictive value of 98%, and a negative predictive value of 100% for a positive response to immunosuppressive therapy.

Myocardial overexpression of TLR4 and HLA-DR, as well as the positivity of circulating cardiac AHA, may be a marker of the response to immunosuppressive therapy.

## 5. Cellular Mechanisms of Cardiac Recovery

The cellular mechanisms of cardiac recovery in patients with inflammatory cardiomyopathy treated with immunosuppression have been investigated in terms of cell death, activation of cell proliferation, and reconstitution of myofibrillar cell contents [52]. Transmission electron microscopy studies showed large cytoplasmic areas that were apparently empty or filled with fine granular material as a result of the reduced myofibrillar content (myofibrillolysis) in all patients at baseline. After 6 months of immunosuppression, the myofibrillar mass and architecture recovered in responders, while in non-responders, the cardiomyocytes showed a further reduction in myofibrillar content. Moreover, the authors analyzed the alpha and beta isoforms of myosin heavy chain (MHC) expression in the responder patients. The increased expression of α-MHC and inhibition of β-MHC synthesis with an enhanced α/β MHC ratio after effective treatment strongly suggested gene activation of fetal protein isoforms that typically become operative in the cell repair process. Apoptotic and necrotic cell death in cardiomyocytes were greater in the baseline biopsies of the responders and non-responders than in the controls, showing that cardiomyocyte loss is an important mechanism of myocardial damage in myocarditis with cardiac dysfunction. The number of cycling myocytes in the baseline myocardial tissue of both the responders and non-responders was greater than in the controls, and it significantly increased after immunosuppression in both groups, suggesting that in chronic myocarditis, there is an activation of myocyte regeneration in order to compensate for cell loss. Therefore, the recovery of cardiac function in patients with myocarditis responding to immunosuppression is associated with inhibition of cell degeneration and death, activation of cell proliferation, and synthesis of new contractile elements.

## 6. Treatment of Specific Types of Immune-Mediated Myocarditis

Specific types of immune-mediated myocarditis require different therapeutic strategies (Table 1).

### 6.1. Eosinophilic Myocarditis

Eosinophilic myocarditis is an uncommon form of myocarditis that is characterized by the presence of patchy, interstitial eosinophilic infiltrates on histology. Eosinophilic myocarditis is generally associated with drug hypersensitivity (i.e., clozapine, carbamazepine, betablockers, clomipramine minocycline, β-lactam antibiotics, and vaccination), autoimmune systemic disorders such as EGPA (formerly Churg–Strauss syndrome), parasitic infections (toxocariasis), hyper-eosinophilic syndrome (HES, idiopathic or clonal), or paraneoplastic events associated with solid tumors, or it can be a primary isolated disease [53]. Also, trauma may cause auto-reactive inflammation of the myocardium and intramural vessels with eosinophilic infiltrates as a consequence of the release of self-antigens following tissue damage caused by trauma [54]. Myocardial hyper-eosinophilic syndrome is characterized by three stages: an acute phase with inflammation and necrosis, a thrombotic phase with subendocardial thrombosis, and a fibrotic end stage evolving versus restrictive cardiomyopathy (Loeffler’s endocarditis). Corticosteroids are a first-line therapy in association with albendazole in Toxocara canis infection, imatinib in myeloproliferative disease, cyclophosphamide, azathioprine or methotrexate in EGPA and HES, and withdrawal of the suspected drug in cases of drug hypersensitivity. In autoreactive myocarditis after chest trauma, immunosuppressive therapy with corticosteroids can determine cardiac recovery. In patients with evidence of intracavitary thrombus, anticoagulation should be initiated. In EGPA and idiopathic HES, anti-IL-5 agents such as mepolizumab [55] and benralizumab [56] are emerging therapies.

### 6.2. Giant Cell Myocarditis

Giant cell myocarditis (GCM) is a rapidly progressing necrotizing myocarditis with a poor prognosis: the rate of death or heart transplant at 3 years is 85% [57]. However, prompt, specific treatment can improve prognoses [58]. On histology, GCM is characterized by extensive infiltration of cytotoxic T cells, macrophages, giant cells, and eosinophils, with massive myocyte necrosis in the absence of granulomas. Myocardial involvement is diffuse; therefore, EMB is a highly sensitive and specific diagnostic tool. GCM equally affects men and women, with a median age between 43 and 53 years [59]. Clinical onset with cardiogenic shock with ventricular tachycardia or complete atrioventricular block is common. Immunosuppressive therapy must be initiated promptly and includes high-dose corticosteroids (methylprednisolone 1 g daily followed by prednisone 1 mg/kg/day tapered gradually, decreasing to 5–10 mg/day after 6–8 weeks, progressively reduced over 1 year then stopped or continued at 5 mg/day) combined with one or more— often two—additional immunosuppressive agents: cyclosporine plus azathioprine, mycophenolate mofetil plus tacrolimus, and/or antithymocyte globulin (ATG) or muromonab CD3 antibody or alemtuzumab plus cyclosporine [60]. Due to mechanical and electrical instability, these patients often require inotropic and mechanical circulatory support, antiarrhythmic treatment, or pacing. In the case of deteriorating cardiogenic shock, a heart transplant is a necessary and effective therapy, with similar survival compared to patients undergoing transplantation for other causes [61]. Nevertheless, the recurrence of GCM can happen in up to 25% of transplant patients and requires aggressive immunosuppression [62]. High-dose corticosteroids and ATG are first-line therapies in patients with recurrent GCM. Sirolimus and rituximab, but above all, alemtuzumab (anti-CD52 antibody), are effective in refractory GCM [63].

### 6.3. Cardiac Sarcoidosis

Cardiac sarcoidosis is a rare systemic disease of unknown etiology that is characterized by non-necrotic inflammatory granulomas that may appear anywhere in the body but commonly involve the lungs and intrathoracic lymph nodes. Cardiac involvement occurs in about 5% of patients and usually presents with cardiogenic shock, acute heart failure, and/or life-threatening arrhythmias [64]. Cardiac sarcoidosis is well-recognized on histology by massive infiltration of macrophages with granulomas and replacement fibrosis responsible for the elevated arrhythmia burden in these patients [65]. The disease distribution is “patchy”, with prevalent localization at the interventricular septum and LV basal free wall, overall conferring a low sensitivity of EMB for diagnosis. Experts’ position statements’ diagnostic criteria for cardiac sarcoidosis are based on positive EMB or extracardiac histological evidence of sarcoidosis with demonstration of cardiac involvement on imaging [66]. 18F-FDG-PET is a key diagnostic tool; a ‘hot spot’ of 18F-FDG overlapping a perfusion defect (“mismatch pattern”) is suggestive of cardiac sarcoidosis [67]. Corticosteroids at relatively high doses are a first-line therapy (methylprednisolone 500–1000 mg/day for 2–3 days followed by prednisone, usually down-titrated every 4 weeks with reductions of 5–10 mg until a maintenance dose of 10 mg/day is reached for 12–16 months) [68]. Methotrexate, azathioprine, mycophenolate mofetil, leflunomide, and cyclophosphamide are often used as second-line therapies in refractory cases or cases of significant steroid side effects. Small studies [69,70] suggest combination therapy from the beginning, but no evidence for improved outcomes exists. Third-line therapies include anti-TNF agents such as infliximab and adalimubab [71]. The Cardiac Sarcoidosis Multi-Center Randomized Controlled Trial (CHASM CS-RCT) is an ongoing, multicenter, randomized, controlled trial designed to compare higher-dose prednisone versus prednisone plus methotrexate [72]. Patients with cardiac sarcoidosis have a 10% risk of sudden cardiac death over 5 years of follow-up [73]. Therefore, according to the 2022 ESC Guidelines for the management of patients with ventricular arrhythmias and the prevention of sudden cardiac death [74], ICD implantation should be considered in patients with cardiac sarcoidosis who have an LVEF > 35% but significant LGE at cardiac MRI after resolution of acute inflammation or LVEF 35–50% and inducible sustained monomorphic ventricular tachycardia at programmed electrical stimulation (PES) (Class of recommendation IIa). Moreover, in patients with cardiac sarcoidosis who have LVEF 35–50% and minor LGE at cardiac MRI after resolution of acute inflammation, PES for risk stratification should be considered (class of recommendation IIa).

### 6.4. ICI-Associated Myocarditis

ICIs are monoclonal antibodies used in numerous types of cancer that target the host immune negative regulation receptors, called CTLA-4 (cytotoxic T-lymphocyte antigen-4), PD-1 (programmed death receptor-1), and its ligand PD-L1 (programmed death-ligand 1), enhancing the T-cell response against cancer. By activating the immune system, ICIs can lead to immune-mediated adverse events such as colitis, dermatitis, and pneumonia [75]. ICI myocarditis, initially described in 2016, is an uncommon but potentially lethal complication of ICIs with a high mortality (50%) [76]. The risk is higher with combination ICI treatment (e.g., ipilimumab and nivolumab). Ventricular arrhythmias and heart failure are common clinical manifestations. On histology, ICI myocarditis is characterized by T-cell and macrophage infiltrates in the myocardium and also in the skeletal muscle, suggestive of lymphocytic myocarditis and myositis [77]. Myositis is common; therefore, troponin and creatine kinase should be monitored during treatment. High-dose corticosteroids associated with withdrawal of ICI are the first-line therapy. Alemtuzumab (anti-CD52 antibody), antithymocyte globulin (anti-CD3 antibody), and abatacept (a CTLA-4 agonist) have been proposed as second-line therapies [78,79].

### 6.5. SARS-CoV-2 mRNA Vaccine-Related Myocarditis

The association between vaccine administration and the onset of myocarditis has been suggested by several case reports and case series [80,81]. The vaccine historically most associated with myocarditis is smallpox [82]. The average age of onset is 25 years, with a greater prevalence in males. Myocarditis usually occurs within 1 week (approximately 3 days) after the second dose [83]. Clinical manifestation is not severe and is self-limiting in most cases. In a US surveillance study, 519 patients with COVID19 vaccine-related myocarditis were followed for 90 days from the onset of symptoms, and more than 90% recovered completely [84]. The physiopathology of SARS-CoV-2 mRNA vaccine-related myocarditis is not clear; molecular mimicry between the spike protein of SARS-CoV-2 and self-antigens has been proposed [85]. EMB was rarely performed, and histology showed inflammatory infiltrates of T cells, macrophages, and eosinophils [86]. Short-term therapies with NSAIDs, colchicine, and steroids have been commonly used in these patients [87,88].

Myocarditis in transplantated hearts is an important form of immune-mediated myocarditis. In general, patients who have undergone organ transplantation are immunosuppressed hosts, leaving them at a higher risk of infections. This aspect is enough to establish a strong predisposition to myocarditis. Moreover, in patients undergoing heart transplantation, recurrency is not uncommon, especially in GCM, and it usually occurs in the first year after heart transplant [61,89]. In most cases, intensification of immunosuppressive protocols is enough to treat GCM recurrence, further contributing to an increased risk of infection and malignancy. Most patients are initially treated with pulse dose methylprednisolone and a prolonged steroid taper. If GCM does not resolve or recurs, more aggressive immunosuppression with ATG can be used. There are also case reports using sirolimus, rituximab, and alemtuzumab. Nonetheless, end-stage graft failure requiring an urgent heart transplant has been described [90].

#### Monitoring and Follow-Up

Uncomplicated forms of myocarditis often resolve spontaneously without relevant sequelae and can be re-evaluated at 6 months from hospital discharge. In cases of complete resolution, follow-up can be suspended after one year. Patients with complicated forms (LV dysfunction or serious arrhythmias at presentation) should be monitored more closely, with assessments of symptoms, inflammatory and cardiac biomarkers, arrhythmias, cardiac function, and tissue characterization at CMR with a timing that mainly depends on their clinical features at discharge [91]. These elements are fundamental for monitoring patients not treated with immunosuppressive therapies and also for evaluating the response to treatment of those in with IS that was started in the acute phase. Moreover, in cases of progressive LV dysfunction or increased VA burden, EMB should be considered to potentially guide further specific treatment. Following a diagnosis of myocarditis, refraining from participation in competitive sports/vigorous exercise is recommended for at least 6 months in all patients, and it should be extended in cases of persistent evidence of myocardial edema or extensive LGE at CMR. Low LVEF at presentation and extensive or anteroseptal LGE at baseline CMR are well-recognized predictors of adverse cardiovascular events in acute myocarditis and should be considered to stratify the longitudinal risk to these patients [92]. From a clinical perspective, is it possible to identify the red flags of immune-mediated pathways suggestive of a beneficial response to immunosuppression in myocarditis patients?

Immunosuppression is the cornerstone of therapy in autoimmune myocarditis. Its role is well-defined in fulminant forms, with common expression of EM and GCMs in lymphocytic virus-negative inflammatory cardiomyopathy, in ICI-associated myocarditis, and in cardiac sarcoidosis. In acute non-fulminant forms, immunosuppression is usually not considered when there is no evidence of concomitant systemic autoimmune diseases. Identification of an immunologic pathway that may benefit from immunosuppressive therapy could be an important source in the management in these patients. There is no evidence of useful clinical, electrocardiographic, or imaging data in this context. Instead, there are specific serological and histological markers of susceptibility to immunosuppression such as the presence of circulating anti-heart autoantibodies and HLA and TLR4 overexpression on the cardiomyocyte surface. Moreover, the knowledge of the role that a reduction in cell death, activation of cell proliferation, and enhancement of cell repair have in the cardiac recovery of inflammatory cardiomyopathy patients receiving immunosuppressive treatment may help to identify new therapeutic strategies. Analysis of new possible serologic and tissue markers in baseline cardiac dysfunction and in follow-up, evaluation of the gene expression profile of the genes implicated in the cardiac reparative processes, and identification of new or unconventional viral agents in non-responders may help to identify new markers of susceptibility to immunosuppression. Further studies are needed in these directions.

**Figure 2:**
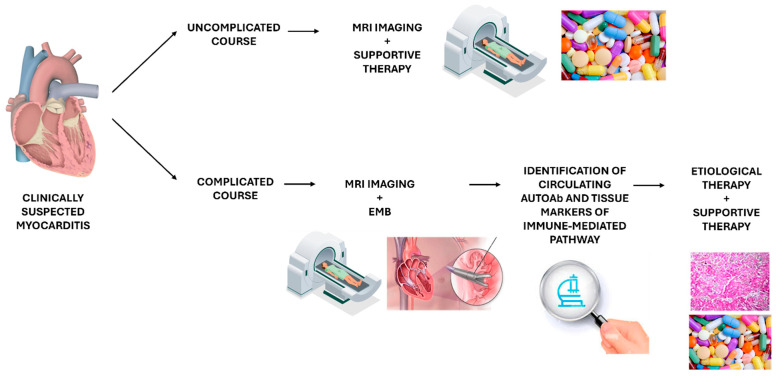
**Diagnostic and therapeutic work-up in immune-mediated myocarditis.** In cases of suspected clinical myocarditis with uncomplicated presentations, the diagnostic and therapeutic work-up may include cardiac MRI imaging and supportive therapy. On the contrary, in cases of complicated myocarditis, cardiac MRI imaging is useful, but EMB is the gold standard to establish etiology and initiate the most appropriate treatment in association with therapy for heart failure and arrhythmias.

Therefore, the need for endomyocardial biopsies in inflammatory cardiomyopathy appears evident, not only for the benefit of patients but also to implement the appropriate diagnostic workup and identify new therapeutic targets. From a clinical point of view, in cases of suspected clinically myocarditis with uncomplicated presentation (e.g., mild symptoms and preserved cardiac function), the diagnostic and therapeutic work-up may include cardiac MRI imaging and supportive therapy. On the contrary, in cases of complicated myocarditis with moderate or severe cardiac dysfunction, cardiogenic shock, or life-threatening arrhythmias, cardiac MRI imaging is useful, but EMB is the gold standard to establish etiology and initiate the most appropriate treatment in association with therapy for heart failure and arrhythmias (Figure 2).

## 7. Conclusions

Myocarditis is an inflammatory disease of the myocardium that is caused by infectious and noninfectious agents. It is still a challenging diagnosis because of its wide variability in clinical presentation and unpredictable course. Immunosuppressive therapy is an important source in the management of virus-negative immune-mediated myocarditis. Identification of immunologic pathways that may benefit from immunosuppressive therapies is fundamental. Further studies are needed.

## Figures and Tables

**Table 1:** Immunosuppressive strategies in virus-negative immune-mediated myocarditis.

Etiology	First-Line Therapy	Second-Line Therapy	Therapy in Fulminant Forms
Lymphocytic (viral, associated with SIDs^7^)	PDN 1 mg/kg/day for 4 weeks followed by 0.33 mg/kg/day for 5 months plus AZA 2 mg/kg/day for 6 months	MMF starting with 1 g/day, then increasing to 2 g/day over 4 weeks (up to 3 g/day if required), plus PDN1 (in cases of AZA^2^ intolerance) plus CP iv 600 mg/m2 at days 1, 15, and 30 in the case of associated SLE or vasculitis	MPDN iv 1 g bolus for 1 or more days, then 1 mg/kg/day to be gradually tapered
Eosinophilic (Immune-mediated, parasitic, drug-related, MPD)	PDN 1 mg/kg/day to be gradually tapered If EGPA, consider CP iv 600 mg/m^2^ at days 1, 15, 30 If toxocariasis, albendazole, 600–800 mg/day, for 2–8 weeks If drug-related withdrawal of suspected drug If associated with MPD: imatinib 100–400 mg/day (up to normalization of eosinophilic count)	MTX 7.5–20 mg weekly or AZA 1–2 mg/kg/day If EGPA or idiopathic HES, consider: Mepolizumab 100–300 mg sc/4 weeks or Benralizumab, 30 mg sc/4–8 weeks	MPDN iv 1 g bolus for 1 or more days, then 1 mg/kg/day to be gradually tapered
Cardiac sarcoidosis	MPDN 500–1000 mg/day for 2–3 days followed by PDN, usually down-titrated every 4 weeks with reductions of 5–10 mg down to 10 mg/day (for 12–16 months)	MTX 15–20 mg/week, AZA 1–2 mg/kg/day, MMF 1–3 g/day or MTX 15–20 mg/week plus infliximab 5 mg/kg or up to 500 mg at time 0 and after 2 and 4 weeks, then every 8 weeks or Adalimubab 40 mg/2 weeks	
Giant cell myocarditis	MPDN 1 g/day followed by PDN 1 mg/kg/day tapered gradually (5–10 mg/day after 6–8 weeks, progressively reduced over 1 year, then stop or continue 5 mg/day) indefinitely combined with one or more of the following: Cy-A target dose 150–300 ng/mL for the first 3 months, 100–150 ng/mL from month 4 to month 12, 75–100 ng/mL thereafter or AZA, 1.5–2 mg/kg/day or MMF, 1.5 g twice daily or Tacrolimus target dose, 10–15 ng/mL in first 6 months, 5–10 ng/mL thereafter or ATG, 100 mg iv daily orMuromonab, 30 mg iv once or 15 mg iv daily for 2 days	High-dose CS with ATG 100 mg iv daily or Alemtuzumab (antiCD52 antibody), single dose of 30 mg plus Cy-A	MPDN iv 10 mg/kg iv bolus plus muromonab 5 mg/day for 10 days
ICI-associated	MPDN 500–1000 mg/day for 2–3 days followed by PDN gradually tapered plus withdrawal of ICI treatment	Alemtuzumab 30 mg single dose, ATG (anti-CD3 antibody) 1 mg/kg single dose or Abatacept 10–25 mg/kg on days 0, 5, and 12	

Legend: PDN, prednisone; AZA, azathioprine; MMF, mycophenolate mofetil; MPDN, methylprednisolone; SIDs, systemic inflammatory disorders; CP, cyclophosphamide; SLE, systemic lupus erythematosus; MTX, methotrexate; EGPA, eosinophilic granulomatosis with polyangiitis; HES, hypereosinophilic syndrome; Cy-A, cyclosporine; ATG, antithymocyte globulin; ICI, immune checkpoint inhibitor.

## Data Availability

The datasets used and analyzed during the current study are avail-able from the corresponding authors upon reasonable request.
